# RACIAL//ETHNIC DIFFERENCES IN WILLINGNESS TO PARTICIPATE IN ALZHEIMER’S DISEASE RESEARCH

**DOI:** 10.1093/geroni/igae098.3702

**Published:** 2024-12-31

**Authors:** Eleanor Skemp, Tanish Sathish, Yichen Zhang, Brian Carpenter

**Affiliations:** Washington University in St. Louis, St. Louis, Missouri, United States; Washington University in St. Louis, St. Louis, Missouri, United States; Washington University in St. Louis, St. Louis, Missouri, United States; Washington University in St. Louis, St. Louis, Missouri, United States

## Abstract

The underrepresentation of minority populations in Alzheimer disease (AD) research has implications for both science and practice, as diversity and inclusion are crucial to ensure generalizable findings and equitable healthcare. However, various factors influence the reluctance of minoritized individuals to participate in AD research, making it important to understand the attitudes that hinder their participation. This study investigated racial/ethnic differences in attitudes about participating in AD research. Results are based on data from a 2022 national survey of U.S. adults (N = 1,274), with a deliberate oversampling of Black respondents. Participants were surveyed about their willingness to join an AD research registry, participate in an AD cohort study, and about reasons underlying their willingness. Participants self-identified as White (47.3%), Black (43.9%), and Hispanic (8.8%). A higher percentage of Blacks were unwilling to participate in a research registry or a cohort study than Whites and Hispanics (p’s <.006), and Blacks were less likely to perceive participation as a contribution to society (p <.001). Hispanics expressed greater concern in cohort studies about the time commitment, the potential to receive negative health news, and fear that research results could be used against them (p’s <.002). As a note of caution, statistically significant differences were relatively small. Despite the limitations a relatively crude racial/ethnic categorization, results could inform AD research recruitment strategies by identifying factors that may cause hesitance among potential participants, with the goal of enhancing the presence of underrepresented groups in AD research.

